# Development and Validation of a Personalized Social Media Platform–Based HIV Incidence Risk Assessment Tool for Men Who Have Sex With Men in China

**DOI:** 10.2196/13475

**Published:** 2019-06-18

**Authors:** Ke Yun, Junjie Xu, Sequoia Leuba, Yunyu Zhu, Jing Zhang, Zhenxing Chu, Wenqing Geng, Yongjun Jiang, Hong Shang

**Affiliations:** 1 Key Laboratory of AIDS Immunology of National Health Commission of the People's Republic of China Department of Laboratory Medicine The First Affiliated Hospital of China Medical University Shenyang China; 2 Key Laboratory of AIDS Immunology of Liaoning Province, The First Affiliated Hospital of China Medical University Shenyang China; 3 Key Laboratory of AIDS Immunology, Chinese Academy of Medical Sciences Shenyang China; 4 Collaborative Innovation Center for Diagnosis and Treatment of Infectious Diseases Hangzhou China; 5 Department of Epidemiology, University of North Carolina Chapel Hill American Samoa; 6 Hebei Yuanqiao Information Technology Co, Ltd Shijiazhuang China

**Keywords:** HIV, risk prediction, social media, men who have sex with men, China

## Abstract

**Background:**

Personalized risk assessments can help medical providers determine targeted populations for counseling and risk reduction interventions.

**Objective:**

The objective of this study was to develop a social media platform–based HIV risk prediction tool for men who have sex with men (MSM) in China based on an independent MSM cohort to help medical providers determine target populations for counseling and risk reduction treatments.

**Methods:**

A prospective cohort of MSM from Shenyang, China, followed from 2009 to 2016, was used to develop and validate the prediction model. The eligible MSM were randomly assigned to the training and validation dataset, and Cox proportional hazards regression modeling was conducted using predictors for HIV seroconversion selected by the training dataset. Discrimination and calibration were performed, and the related nomogram and social media platform–based HIV risk assessment tool were constructed.

**Results:**

The characteristics of the sample between the training dataset and the validation dataset were similar. The risk prediction model identified the following predictors for HIV seroconversion: the main venue used to find male sexual partners, had condomless receptive or insertive anal intercourse, and used rush poppers. The model was well calibrated. The bootstrap C-index was 0.75 (95% CI 0.65-0.85) in the training dataset, and 0.60 (95% CI 0.45-0.74) in the validation dataset. The calibration plots showed good agreement between predicted risk and the actual proportion of no HIV infection in both the training and validation datasets. Nomogram and WeChat-based HIV incidence risk assessment tools for MSM were developed.

**Conclusions:**

This social media platform–based HIV infection risk prediction tool can be distributed easily, improve awareness of personal HIV infection risk, and stratify the MSM population based on HIV risk, thus informing targeted interventions for MSM at greatest risk for HIV infection.

## Introduction

### Background

A significant global challenge to HIV prevention and control is the HIV epidemic concentrated among men who have sex with men (MSM) [1]. In China, the annual reported incident HIV/AIDS cases among MSM is very high. In 2015, the HIV prevalence among MSM was 8%, and the HIV incidence among MSM was 5.61 cases per 100 person-years [2]. To end the global HIV epidemic by 2030, the Joint United Nations Programme on HIV and AIDS (UNAIDS) developed the 90-90-90 strategy, and the first “90” goal is to have 90% of HIV-infected people know their HIV serostatus by 2020 [3]. However, an estimated 50% of HIV-infected Chinese MSM do not know their serostatus [4], and only 60.5% of Chinese MSM have ever been tested for HIV in their lifetime [5]. This low HIV testing rate among Chinese MSM could be caused by an inaccurate evaluation of their personal HIV infection risk. A study examining perceived risk and HIV acquisition found that over 50% of participants diagnosed with HIV in the study thought their lifelong risk of HIV infection was very low or none [6].

Given that the risk of HIV infection among MSM varies, identifying MSM who are at higher risk of HIV seroconversion can improve targeted prevention interventions, which include HIV screening and preexposure prophylaxis (PrEP) [7]. In recent years, prospective cohort studies have examined HIV seroconversion predictors among MSM, including multiple sexual partners, condomless anal intercourse, sexually transmitted infections, and rush poppers usage [8]. Rush poppers are an inhalable mixture of various nitrites that are frequently used among the MSM community as they can relax smooth muscle, expand peripheral blood vessels, and relieve pain during anal sex, and thus increase sexual pleasure [8]. Insight into these predictors can lead to more accurate predictions of HIV seroconversion risk among MSM. Several risk evaluation models specific to MSM have been developed to quantify this risk including the Denver model [9], the University of North Carolina at Malawi Risk Screening Score model [10], and the San Diego Early Test (SDET) score model [11]. In addition, prediction models specific to Chinese MSM have been developed to estimate HIV infection risk [12,13]. However, all the previous models mentioned were developed using cross-sectional survey data, and thus could not predict longitudinal HIV seroconversion risk. The Menza score is a risk prediction model that could predict the risk of longitudinal HIV seroconversion because it used prospective cohort data [14]. However, the data were derived from MSM in the United States, and because of differences in the distribution of social and cultural demographics and risk factors among MSM, this model is not directly applicable to MSM in China [14].

Social media is an important communication tool to build virtual communities and networks, especially among the MSM community. WeChat is the most popular messaging and social media app in China; it had over 1 billion monthly active users in 2018 [15]. An online survey conducted among Chinese MSM reported that 57.9% of participants used online dating apps, which are associated with risky sexual behaviors and may foster a virtual environment that increases the risk of sexually transmitted diseases [16]. However, most published HIV risk prediction models for MSM are circulated through webpages and not through newer social media platforms.

### Objectives

This study aims to build a prospective cohort-derived longitudinal HIV seroconversion risk assessment tool that is based on a popular social media platform to provide easy access for MSM to determine their personalized HIV incidence risk and inform targeted interventions.

## Methods

### Study Design and Participants

The open prospective cohort enrolled MSM from Shenyang, China, through a snowball sampling method and followed this cohort from January 2009 to January 2016 through the voluntary counseling and testing center in the First Affiliated Hospital of China Medical University [17]. The MSM cohort was recruited from baths, bars, a social media app for the gay community, and other venues as conducted by community-based organization leaders. Initial participants were asked to recruit partners or peers to participate in the survey and given 50 yuan (approximately US $8) for each recruited participant. Following written informed consent, eligible participants were interviewed face-to-face by a trained staff member in a private counseling room. The interview asked about demographics, sexual practices, and substance use, including the history of recreational drug use and whether the participant used poppers and/or methamphetamine in the past 3 months. Condoms and lubricants were freely distributed to each MSM participant. Both HIV-1 and syphilis tests were conducted on each participant. Pretest and posttest counseling were provided to each participant at both the baseline and follow-up HIV-1 and syphilis tests. The cohort inclusion criteria were the following: (1) aged 15 years or older, (2) male who self-reported anal and/or oral intercourse experience with a male partner in the past 6 months, (3) baseline negative HIV antibody and nucleic acid screening, and (4) informed consent signed by themselves or their guardian. The study participants were assigned a unique six-digit personal identification code, which was used to link their test results to demographic information.

### Potential Risk Factors of HIV Incidence

Factors that may be associated with HIV seroconversion among MSM in this cohort include demographic characteristics, sexual practices, sexual role, condom use during anal intercourse, number of male sexual partners, and history of recreational drug use. We used a recall window of the past 3 months. We used the HIV/AIDS-related knowledge questionnaire, which consisted of eight questions, and 1 point was given for the correct answer to each question. The questionnaire was the following:

1. Is it possible for a healthy person to be infected with HIV?
2. Can blood or blood products with HIV virus spread HIV?
3. Can sharing needles with others transmit HIV infection?
4. Can condom usage reduce the risk of HIV infection?
5. Does having a monogamous HIV-negative partner decrease the risk of HIV acquisition?
6. Can HIV be transmitted from an HIV-positive pregnant woman to her baby?
7. Can HIV be transmitted through having dinner with an HIV-positive individual?
8. Can HIV be spread through mosquito bites? [18]

We defined the outcome of HIV seroconversion as a baseline HIV antibody seronegative case that seroconverted to an HIV antibody seropositive case during the follow-up period. We defined the HIV seroconversion time as the midpoint date between the last tested HIV-seronegative date and the first tested HIV-seropositive date.

### Laboratory Testing

Both HIV and syphilis testing were performed every 3 months during follow-up. The HIV screening test was performed through enzyme-linked immunosorbent assay (ELISA), and suspected positive cases were further confirmed by Western blot. HIV-1 antibody-negative cases and positive cases in which Western blot was uncertain or negative were further confirmed negative through pooled real-time polymerase chain reaction (Cobas Amplicor HIV-1 MONITOR Tom Test, v1.5, Roche, 21118390123). Syphilis serology was performed using the rapid plasma reagin test (RPR; Shanghai Kehua, China), and positive cases were further confirmed by the Treponema pallidum particle assay (TPPA, Serodia, Japan). Participants with plasma positive for both RPR and TPPA were deemed to be currently infected with syphilis.

### Statistical Analysis and WeChat Applet Construction

The overall dataset was randomly divided into training and validation datasets at the approximate ratio of 2:1. Variables were selected using the backward variable selection method based on the Akaike information criterion in the Cox regression model. Variables with a *P* value less than .25 in the univariable Cox regression model were entered into the multivariable regression for variable selection, and variables with a *P* value less than .05 were retained in the final model. We used a bootstrap resampling procedure of 10,000 samples to test the stability of the predictive score in the training and validation datasets. We also used the Cox.zph function in the “survival” package of R to test the validity of the proportional hazards assumption. Internal and external consistency of the discrimination and calibration performance measures were evaluated by the bootstrap resampling procedure. Discrimination was evaluated using Harrell’s concordance statistic (C-index), and calibration was conducted by comparing the actual proportion of those without incident HIV infection with the predicted probability of no incident HIV infection developed from the Kaplan-Meier estimates for each decile. We used multiple imputation methods to address missing values. If the variable had more than 50% missing, it was discarded. Based on the predictive model with the identified risk factors, a nomogram of the prediction model was constructed to allow visual estimation of the predicted 2- and 4-year cumulative risk. Alternatively, the risk of HIV infection could be calculated using a WeChat-based HIV risk calculator. The WeChat-based HIV risk calculator was designed as follows: (1) the system adopts client-server structure to communicate with transmission control protocol and the Internet Protocol protocol; (2) the server adopts model-view-controller design and the client is developed by WeiXin Markup Language, JavaScript based on the WeChat platform; (3) build relational database MySQL for data storage; and (4) build and run an HIV risk assessment software program on a WeChat applet compatible with iOS and Android mobile devices. We developed three stratified risk subgroups using the tertial cutoff points of the linear prediction value for risk stratification [19]. The cumulative incidences of HIV seroconversion among the three risk subgroups were compared by the log-rank test. The predicted 2- and 4-year absolute risks were calculated from the baseline probability, and the relative risk profile was developed from the Cox proportional hazards regression model. All statistical analyses were performed using SAS 9.4 (Cary, NC, USA) and R software version 2.13.2. A two-sided *P* value of less than .05 was considered statistically significant. The study protocol was approved by the Institutional Review Board of the First Affiliated Hospital of China Medical University, Shenyang, China ([2011]-36).

## Results

### Selection and Characteristics of the Cohort

**Figure 1:**
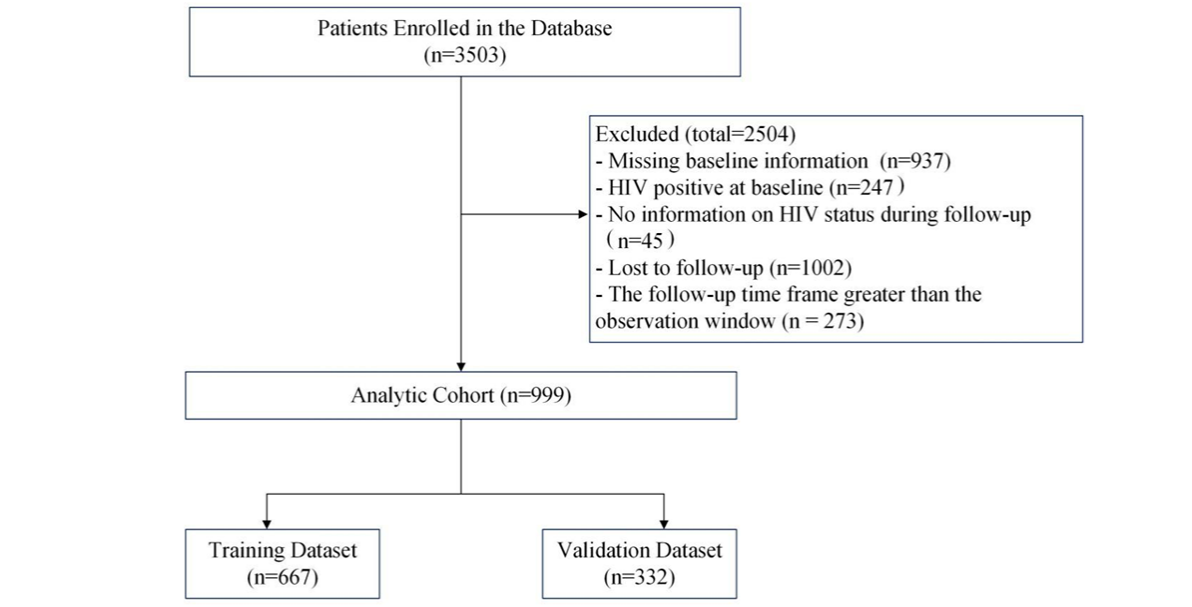
Flowchart of study selection criteria from a prospective cohort of men who have sex with men from 2009 to 2016 in Shenyang, China.

We examined 3503 medical records from a prospective open cohort of MSM followed from January 2009 to January 2016 in Shenyang, China. After excluding participants who had no follow-up data or were HIV positive at baseline, 999 MSM were included for the model construction. Of these, 667 MSM were randomly placed in the training dataset, and 332 were randomly placed in the validation dataset (Figure 1).

In the overall dataset, the mean age of the MSM was 27.5 (SD 9) years, and the range was 15 to 68 years. Overall, 48.7% (487/999) were aged 24 years or younger, 65.1% (650/999) had a local household registration, 85.8% (857/999) were ethnically Han, 65.3% (652/999) had a monthly income of less than US $430, 75.3% (752/999) were single, 39.8% (398/999) had a college and above education level, and 55.9% (558/999) had an AIDS knowledge score of less than 8 points. There were no statistically significant differences between the characteristics of the overall dataset, the training dataset, and the validation dataset, suggesting that the randomization into each subset worked well (Table 1).

**Table 1:** Characteristics of MSM participants in the overall, training, and validation dataset.

Characteristics and subgroup		Overall dataset (N=999), n (%)	Training dataset (n=667), n (%)	Validation dataset (n=332), n (%)	*P* value
**Age (years)**					.42
	≤24	487 (48.7)	317 (47.5)	170 (51.2)	
	>24	512 (51.3)	350 (52.5)	162 (48.8)	
**Local residence**					.67
	Yes	650 (65.1)	431 (64.6)	219 (66.0)	
	No	349 (34.9)	236 (35.4)	113 (34.0)	
**Ethnicity**					.82
	Non-Han	142 (14.2)	96 (14.4)	46 (13.9)	
	Han	857 (85.8)	571 (85.6)	286 (86.1)	
**Monthly income (US$)**					.54
	<430	652 (65.3)	431 (64.6)	221 (66.6)	
	≥430	347 (34.7)	236 (35.4)	111 (33.4)	
**Education**					.81
	Less than high school	342 (34.2)	224 (33.6)	118 (35.5)	
	High school	259 (25.9)	176 (26.4)	83 (25.0)	
	College and above	398 (39.8)	267 (40.0)	131 (39.5)	
**Marital status**					.48
	Single	752 (75.3)	507 (76.0)	245 (73.8)	
	Married or cohabiting with a partner	247 (24.7)	160 (24.0)	87 (26.2)	
**AIDS knowledge scores**					.64
	<8	558 (55.9)	376 (56.4)	182 (54.8)	
	8	441 (44.1)	291 (43.6)	150 (45.2)	
**Age of sexual debut with males (years)**					.58
	<30	917 (91.8)	610 (91.5)	307 (92.5)	
	≥30	82 (8.2)	57 (8.5)	25 (7.5)	
**Main venue used to seek male sexual partners**					.32
	Internet	527 (52.8)	360 (54.0)	167 (50.3)	
	Bars/dance halls	43 (4.3)	31 (4.6)	12 (3.6)	
	Parks/public baths	429 (42.9)	276 (41.4)	153 (46.1)	
**Had regular male sexual partners**					.59
	Yes	571 (57.2)	385 (57.7)	186 (56.0)	
	No	428 (42.8)	282 (42.3)	146 (44.0)	
**Had casual male sexual partners**					.74
	Yes	597 (59.8)	396 (59.4)	201 (60.5)	
	No	402 (40.2)	271 (40.6)	131 (39.5)	
**Number of male sexual partners**					.93
	<3	612 (61.3)	408 (61.2)	204 (61.4)	
	≥3	387 (38.7)	259 (38.8)	128 (38.6)	
**Had condomless insertive anal intercourse**					.52
	Yes	384 (38.4)	261 (39.1)	123 (37.0)	
	No	615 (61.6)	406 (60.9)	209 (63.0)	
**Had condomless receptive anal intercourse**					.51
	Yes	351 (35.1)	239 (35.8)	112 (33.7)	
	No	648 (64.9)	428 (64.2)	220 (66.3)	
**Rush poppers use**					.92
	Yes	100 (10.0)	65 (9.7)	35 (10.5)	
	No	538 (53.9)	361 (54.1)	177 (53.3)	
	Not available	361 (36.1)	241 (36.1)	120 (36.1)	
**Tested positive for syphilis**					.55
	Yes	81 (8.1)	57 (8.5)	24 (7.2)	
	No	685 (68.6)	450 (67.5)	235 (70.8)	
	Not tested	233 (23.3)	160 (24.0)	73 (22.0)	

### Cox Regression Analysis and Risk Score Formula

Table 2 lists the independent predictors for HIV seroconversion among MSM with the hazard ratios calculated by the multivariable Cox proportional hazards regression model. The hazard was lower for having condomless insertive anal intercourse (beta=−1.51, adjusted hazard ratio [aHR]=0.22, 95% CI 0.09-0.55, *P*=.001) compared to not having condomless insertive anal intercourse. The hazard was higher for having condomless receptive anal intercourse (beta=1.10, aHR=3.01, 95% CI 1.48-6.16, *P*=.003) compared to not having condomless receptive anal intercourse. The hazards were higher for either using the internet as the main venue to seek male sexual partners (beta=0.99, aHR=2.70, 95% CI 1.02-7.14, *P*=.046) or for using bars or dance halls as the main venue to seek male sexual partners (beta=2.06, aHR=7.84, 95% CI 2.64-22.91, *P*=.03) compared to using parks or public baths as the main venue to seek male sexual partners, and for rush poppers use (beta=0.88, aHR=2.40, 95% CI 1.10-5.27, *P*=.03) compared to not using rush poppers. The HIV prediction Cox regression model was thus the following:

*F*(*t*)=1−[*S*_0_(*t*)]^exp[(−1.51)×^^x^^1+1.10×^^x^^2+0.99×^^x^^3^^a^^+2.06×^^x^^3^^b^^+0.88×^^x^^4)])^

where *F(t)* is the risk function or the probability of incident HIV infection over time (in years), *S*_0_*(t)* is the baseline survival function; *x1* is had condomless insertive anal intercourse (0=no, 1=yes), *x2* is had condomless receptive anal intercourse (0=no, 1=yes), *x3a* is the dummy variable that the main venue used to seek male sexual partners is the internet (1=internet, 0=parks/public baths), and *x3b* is the dummy variable that the main venue used to seek male sexual partners is bars or dance halls (1=bars/dance halls, 0=parks/public baths), and *x4* is rush poppers use (0=no, 1=yes). From the Kaplan-Meier estimation, the HIV-uninfected probabilities, *S*_0_*,* were calculated as 0.96 for baseline, 0.90 for 1 year, 0.86 for 2 years, 0.80 for 3 years, and 0.74 for 4 years.

### Model Discrimination and Calibration

**Figure 2:**
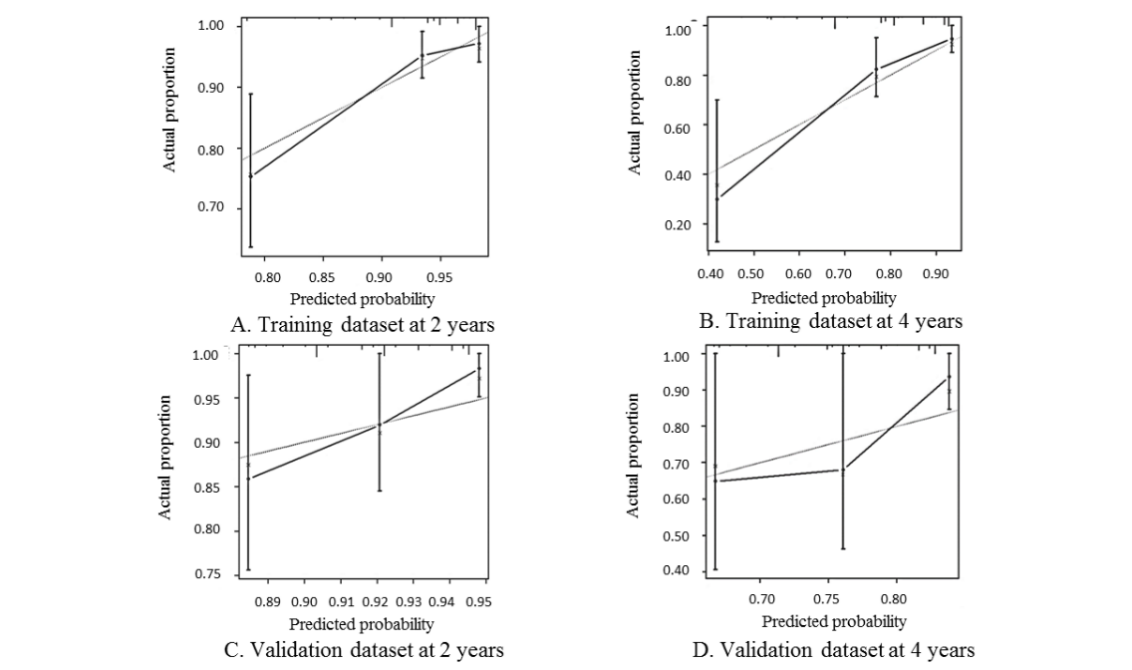
Calibration plot of the HIV seroconversion risk prediction model for the training dataset at (A) 2 years and (B) 4 years, and for the validation dataset at (C) 2 years and (D) 4 years. The x-axes present the predicted probability of no incident HIV infection; the y-axes present the actual probability of no incident HIV infection. The gray lines are the actual proportion of men who have sex with men without incident HIV infection, the black lines are predicted probabilities, and the vertical capped lines are estimates with the 95% confidence intervals.

The C-index was 0.75 (95% CI 0.65-0.85) in the training dataset, and 0.60 (95% CI 0.45-0.74) in the validation dataset. The calibration plot for the probability of no HIV infection at 2 years and at 4 years showed good agreement between predicted risk and the actual proportion of no HIV infection in both the training and validation datasets (Figure 2).

**Table 2:** Cox regression analysis of HIV seroconversion among men who have sex with men in the training dataset.

Characteristics and subgroup		Univariate analysis		Multivariate analysis	
		cHR^a^ (95% CI)	*P* value	aHR^b^ (95% CI)	*P* value
**Age (years)**					
	≤24	1.00			
	>24	0.98 (0.62-1.54)	.92	—^c^	—
**Local residence**					
	Yes	1.00			
	No	0.97 (0.61-1.54)	.89	—	—
**Ethnicity**					
	Non-Han	1.00			
	Han	1.09 (0.58-2.07)	.78	—	—
**Monthly income (US$)**					
	<430	1.00			
	≥430	0.89 (0.70-1.14)	.36	—	—
**Education**					
	Less than high school	1.00			
	High school	0.72 (0.40-1.31)	.29	—	—
	College and above	1.09 (0.65-1.84)	.75	—	—
**Marital status**					
	Single	1.00			
	Married or cohabiting with a partner	0.50 (0.27-0.91)	.02	—	—
**AIDS knowledge scores**					
	<8	1.00			
	8	0.86 (0.53-1.39)	.54	—	—
**Age of sexual debut (years)**					
	< 30	1.00			
	≥ 30	0.14 (0.02-0.98)	.02	—	—
**Main venue used to seek male sexual partners**					
	Internet	1.81 (1.08-3.04)	.02	2.70 (1.02-7.14)	.046
	Bars/dance halls	5.58 (2.09-14.93)	<.001	7.84 (2.64-22.91)	.03
	Parks/public baths	1.00			
**Number of male sexual partners**					
	<3	1.00			
	≥3	2.06 (1.29-3.28)	.002	—	—
**Had condomless insertive anal intercourse**					
	Yes	0.66 (0.41-1.07)	.09	0.22 (0.09-0.55)	.001
	No	1.00		1.00	
**Had condomless receptive anal intercourse**					
	Yes	1.60 (1.00-2.50)	.047	3.01 (1.48-6.16)	.003
	No	1.00		1.00	
**Rush poppers use**					
	Yes	2.34 (1.10-5.00)	.004	2.40 (1.10-5.27)	.03
	No	1.00		1.00	
**Tested positive for syphilis**					
	Yes	1.59 (0.85-2.98)	.15	—	—
	No	1.00			

^a^cHR: crude hazard ratio.

^b^aHR: adjusted hazard ratio.

^c^Not applicable.

### Development of the HIV Risk Assessment Tools

**Figure 3:**
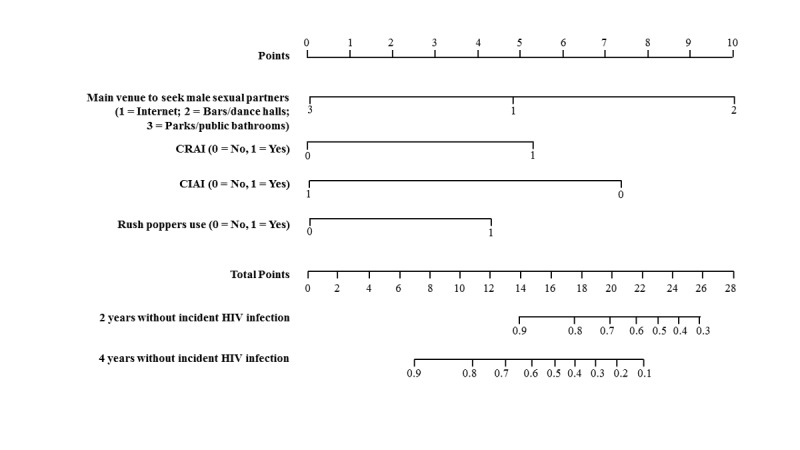
Nomogram for incident HIV infection among men who have sex with men. To use the nomogram, the health official first determines and then sums the points of each variable located on the top point scale. Based on this sum, the health official uses the bottom point scale to determine the probability of HIV incidence. CIAI: condomless insertive anal intercourse; CRAI: condomless receptive anal intercourse.

To facilitate the application of our model by public health workers and by the MSM community, we constructed a nomogram and a social media platform–based calculator. A nomogram is a visual risk assessment tool that allows for the approximate graphical computation of a mathematical function, and it integrates all the significant independent factors for HIV seroconversion determined by the training dataset to predict HIV seroconversion risk at multiple time points in the future (Figure 3). As WeChat is the most popular social media app in China, we also developed a WeChat-based risk calculator that is accessible through a QR code to facilitate distribution of our risk prediction model (Multimedia Appendix 1). Using the nomogram or the WeChat-based risk calculator, MSM individuals can enter their own HIV-related behavioral characteristics and quickly learn their objective risk of HIV infection in the future.

**Figure 4:**
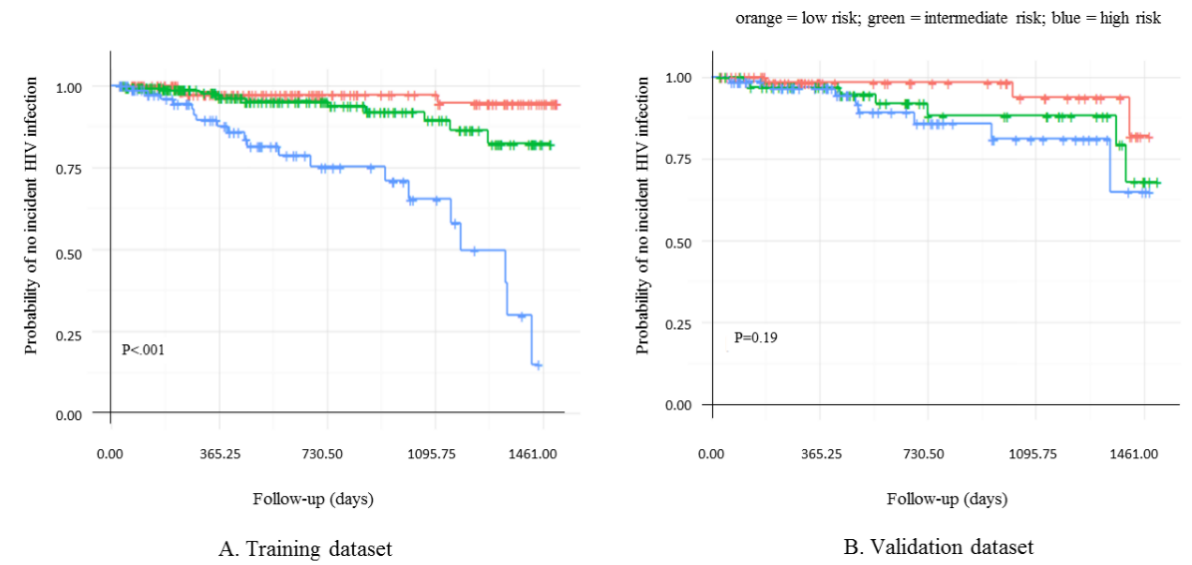
Risk stratification through Kaplan-Meier survival curves for (A) the training dataset and (B) the validation dataset.

To facilitate the use of these two tools, tertile cutoff points of the linear predictor of risk based on the Cox regression were used to stratify MSM into low-, intermediate-, or high-risk subgroups, and the cutoff points were determined at 0.10 and 0.77. The log-rank test indicated a significant difference for the probability of no incident HIV infection among the three subgroups in the training dataset (*P*<.001), and a different trend among three subgroups in the validation dataset (*P*=.19; Figure 4).

## Discussion

### Principal Findings

This study constructed an HIV incidence risk assessment model based on data from an open prospective MSM cohort and developed two tools to convey this personalized HIV acquisition risk (ie, the nomogram and WeChat-based risk calculator). These tools can provide MSM their accurate objective risk of incident HIV infection over time and thus can stratify MSM into distinct risk subgroups (ie, low, intermediate, or high risk). The WeChat applet was built and distributed through the most popular social media platform in China to facilitate the use of this tool.

Our study found that rush poppers use is an independent predictor for HIV seroconversion among MSM. Studies have reported that rush poppers use was associated with sexual behaviors that have a high risk of HIV infection, such as multiple sexual partners, commercial sex, and group sex [20]. Thus, our inclusion of this risk factor for HIV seroconversion in our model is concurrent with previous findings. Similar to the Menza model which used Seattle & King County STD clinic electronic records to estimate hazard ratios for HIV acquisition based on Cox proportional hazards models, unprotected anal intercourse with a partner of positive or unknown HIV status as the acknowledged predictors of HIV seroconversion in Menza score [14], we also find this predictor to be significant in our multivariable analysis. Therefore, partners of individuals found to be HIV positive should be notified promptly by the health department and offered testing and treatment to diagnose and reduce secondary HIV transmission [21].

We also found that compared to the main venue for seeking male sexual partners being parks or public baths, the main venue being bars or clubs or being the internet were independent predictors of HIV seroconversion among MSM. The Guangdong HIV prediction model found that MSM among different venues (ie, parks, public baths, bars/clubs, or internet) had different high-risk sexual behaviors and HIV infection risks [12]. For example, since sex workers often recruit customers at bars or clubs, MSM who use bars or clubs as their main venue to seek male sexual partners may have more commercial sex [22]. In addition, MSM who use bars or clubs as their main venue to seek male sexual partners are younger compared to MSM who use parks or public baths [23]. Therefore, to efficiently address the HIV epidemic among young MSM, HIV prevention strategies must be implemented in bars or clubs. Moreover, since male sexual partners are more readily accessible because of the internet and social media facilitating connections, MSM who use the internet to seek male sexual partners have more sexual partners and unprotected anal intercourse [24-28] than MSM who use other venues to find male sexual partners. Thus, researchers should take advantage of the internet and social media to advertise and distribute tailored comprehensive intervention packages.

Previous models used odds ratios as the scoring value; whereas, we used hazard ratios. Compared to odds ratios, which are cumulative over a time span with a defined endpoint, hazard ratios represent the instantaneous risk over the study period, and thus are easier to understand and interpret by clinicians and the MSM community. In addition, our tool, in contrast to previously published risk assessment tools, can predict the incident HIV infection risk in the next 1, 2, 3, and 4 years.

Although the discriminatory accuracy of our model was modest, the C-index estimates that we report are slightly higher than those of other risk models, such as the Menza score [14], SDET score [11], which were also commonly used to guide clinical decisions. This cumulative risk evaluation over time accurately provides MSM the impact of their high-risk sexual behaviors on their personal HIV infection risk. Finally, our tools can be applied in other settings and countries based on our methods. Other researchers could use their local MSM prospective cohort data to calculate their own parameters to develop their specialized HIV risk assessment tool. In addition, researchers in countries with a large social media presence should also consider advertising and distributing these tools through popular social media platforms.

We constructed a personalized and objective model, and further developed a nomogram, a graphical calculating device widely used in tumor prediction models [29,30], and a WeChat-based HIV risk assessment, which can be used on mobile phones or tablets to facilitate the distribution and application of our model to the MSM community. We designed our tools to be easily accessible and distributed through a social media platform compared with traditional internet-based HIV prediction tools [31]. We based this tool on the most popular social media platform in China, WeChat, because MSM frequently use this platform to communicate, seek sexual partners, and share information and thus can reach all members of the MSM social network [32,33]. In addition, the WeChat applet is compatible with iOS and Android operating systems, and our risk prediction tool can be easily combined with the existing WeChat functions facilitating online and offline HIV prevention and treatment. Our WeChat risk prediction app is readily accessible and the MSM community can easily determine their personalized HIV infection risk and be connected to comprehensive health interventions [34]. Since 2017, we recorded 4158 visits to our WeChat applet. We are currently conducting a randomized controlled clinical trial to assess the effectiveness of this HIV risk prediction tool at promoting HIV testing and decreasing HIV-related high-risk behaviors, such as reducing the number of sexual partners, increasing the proportion of condom usage, etc. This online comprehensive intervention based on the HIV risk prediction model could reduce the number of homosexual partners and promote the use of condoms with casual partners in the MSM population.

### Limitations

Our WeChat tool can only be used through WeChat, and thus is only available in China and neighboring countries. However, as our model is based on a cohort of Chinese MSM, we expect our tool to be most accurate when applied in China. Second, the current HIV infection risk assessment software gives related prevention and control recommendations but is not connected to more substantive HIV interventions. Future steps include integrating this risk assessment tool with other ongoing HIV detection and HIV high-risk behavior intervention projects for MSM to improve risk perception and promote regular HIV testing and HIV prevention services. We also used snowball sampling and thus may have sampling bias as the social network collected is not random and may be limited to a specific group or geographic area. Finally, the recruitment time period spans 8 years and characteristics of the MSM community and risk factors of HIV infection may have changed over this time period; therefore, our results may be less accurate than a larger cohort followed for a shorter period of time.

### Conclusions

We developed and validated an HIV incidence risk assessment model for MSM in China. This model provides objective self-assessments and predictions of incident HIV infection risk. We then developed this model into two separate tools with one tool built on the most popular social media platform in China. MSM can use these tools to quantify their personal HIV infection risk. In addition, public health officials and community health workers can use these tools to conduct accurate quantitative HIV risk assessments of their MSM patients and thus determine HIV high-risk participants to target for risk reduction interventions in hopes of mitigating the HIV epidemic among MSM.

## Appendix

Multimedia Appendix 1 Risk prediction WeChat applet instructions and architecture.

## Abbreviations

aHR: adjusted hazard ratio
cHR: crude hazard ratio
ELISA: enzyme-linked immunosorbent assay
MSM: men who have sex with men
PrEP: preexposure prophylaxis
RPR: rapid plasma reagin test
TPPA: Treponema pallidum particle agglutination assay
SDET: San Diego Early Test
