# Supplementary material for: Development and Validation of a Personalized Social Media Platform–Based HIV Incidence Risk Assessment Tool for Men Who Have Sex With Men in China
Source: J Med Internet Res. 2019 Jun 18;21(6):e13475. doi: 10.2196/13475 (PMC6604506; doi:10.2196/13475)
Supplement: Multimedia Appendix 1 [file jmir_v21i6e13475_app1.pptx]

## Slide 1
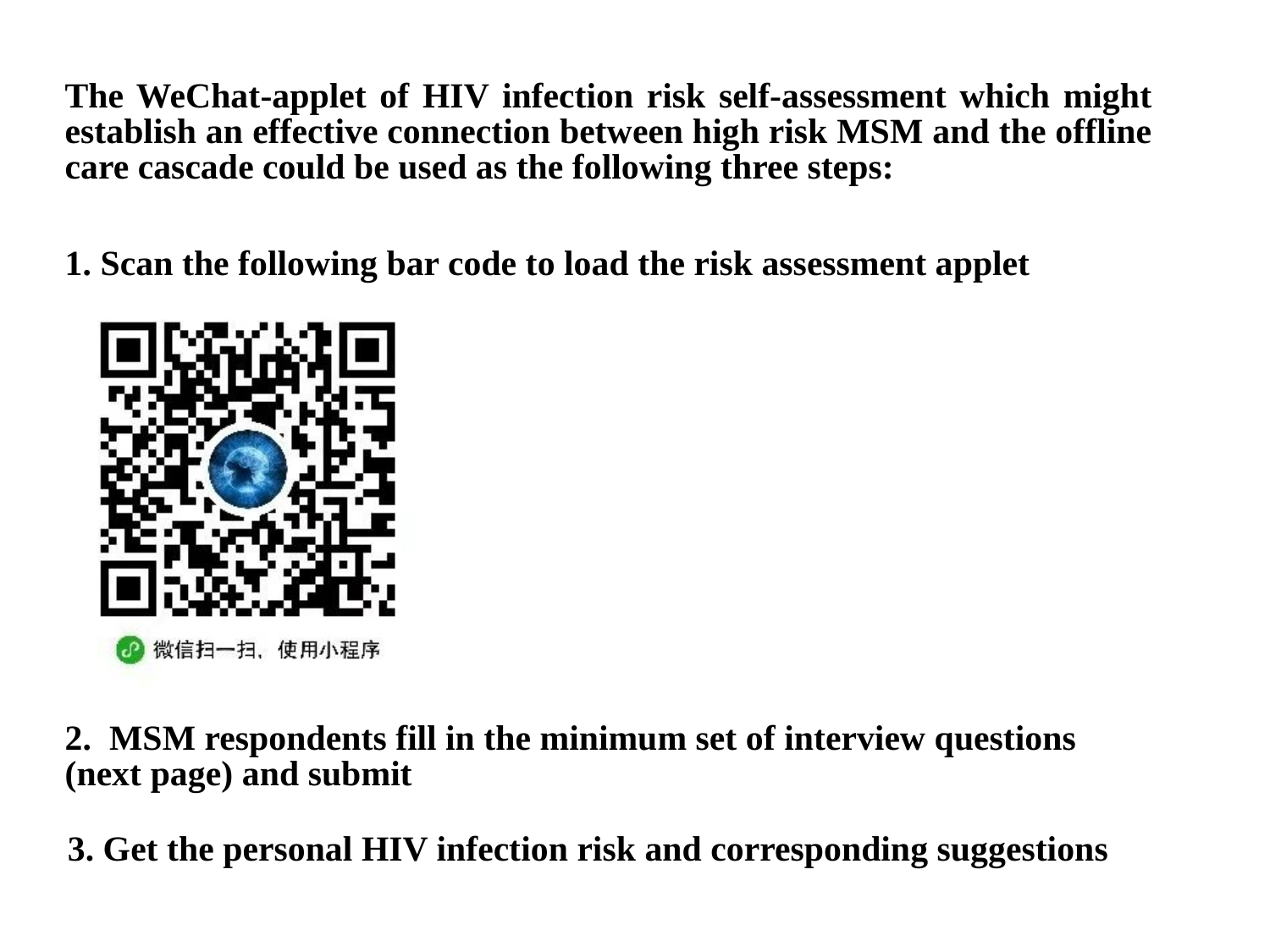

The WeChat-applet of HIV infection risk self-assessment which might establish an effective connection between high risk MSM and the offline care cascade could be used as the following three steps:
1. Scan the following bar code to load the risk assessment applet
2. MSM respondents fill in the minimum set of interview questions (next page) and submit
3. Get the personal HIV infection risk and corresponding suggestions

## Slide 2
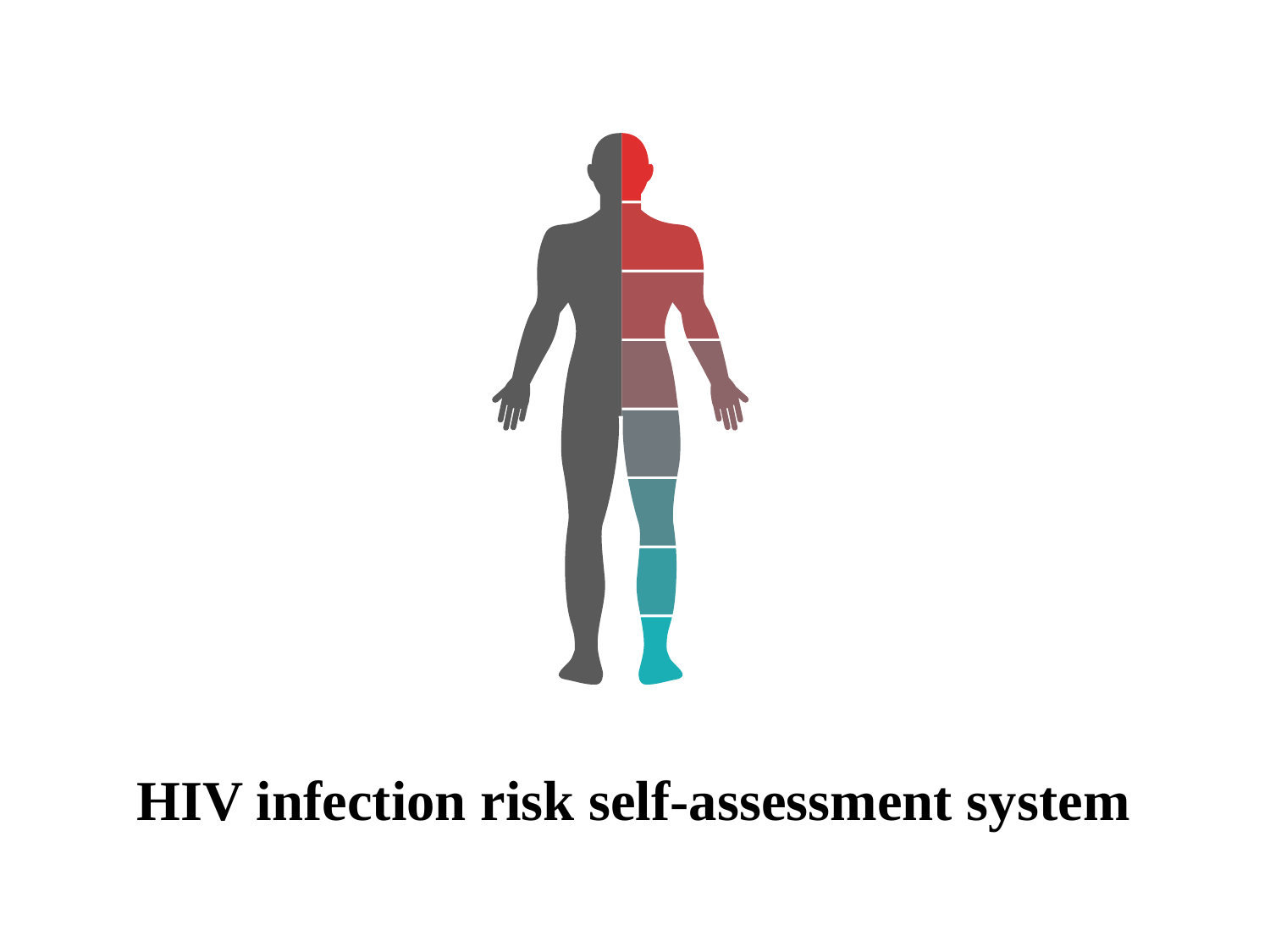

HIV infection risk self-assessment system

## Slide 3
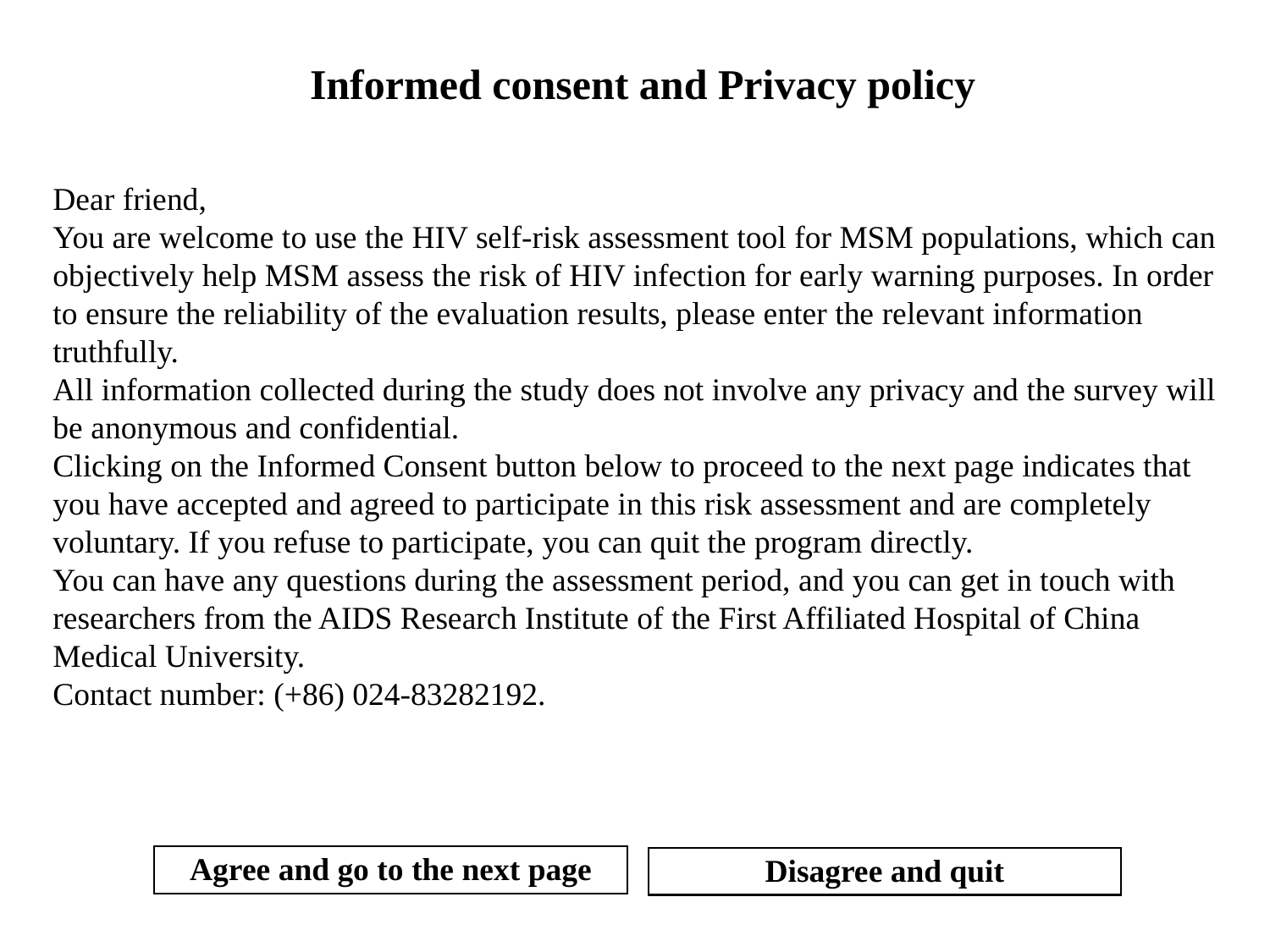

Informed consent and Privacy policy
Dear friend,
You are welcome to use the HIV self-risk assessment tool for MSM populations, which can objectively help MSM assess the risk of HIV infection for early warning purposes. In order to ensure the reliability of the evaluation results, please enter the relevant information truthfully.
All information collected during the study does not involve any privacy and the survey will be anonymous and confidential.
Clicking on the Informed Consent button below to proceed to the next page indicates that you have accepted and agreed to participate in this risk assessment and are completely voluntary. If you refuse to participate, you can quit the program directly.
You can have any questions during the assessment period, and you can get in touch with researchers from the AIDS Research Institute of the First Affiliated Hospital of China Medical University.
Contact number: (+86) 024-83282192.
Agree and go to the next page
Disagree and quit

## Slide 4
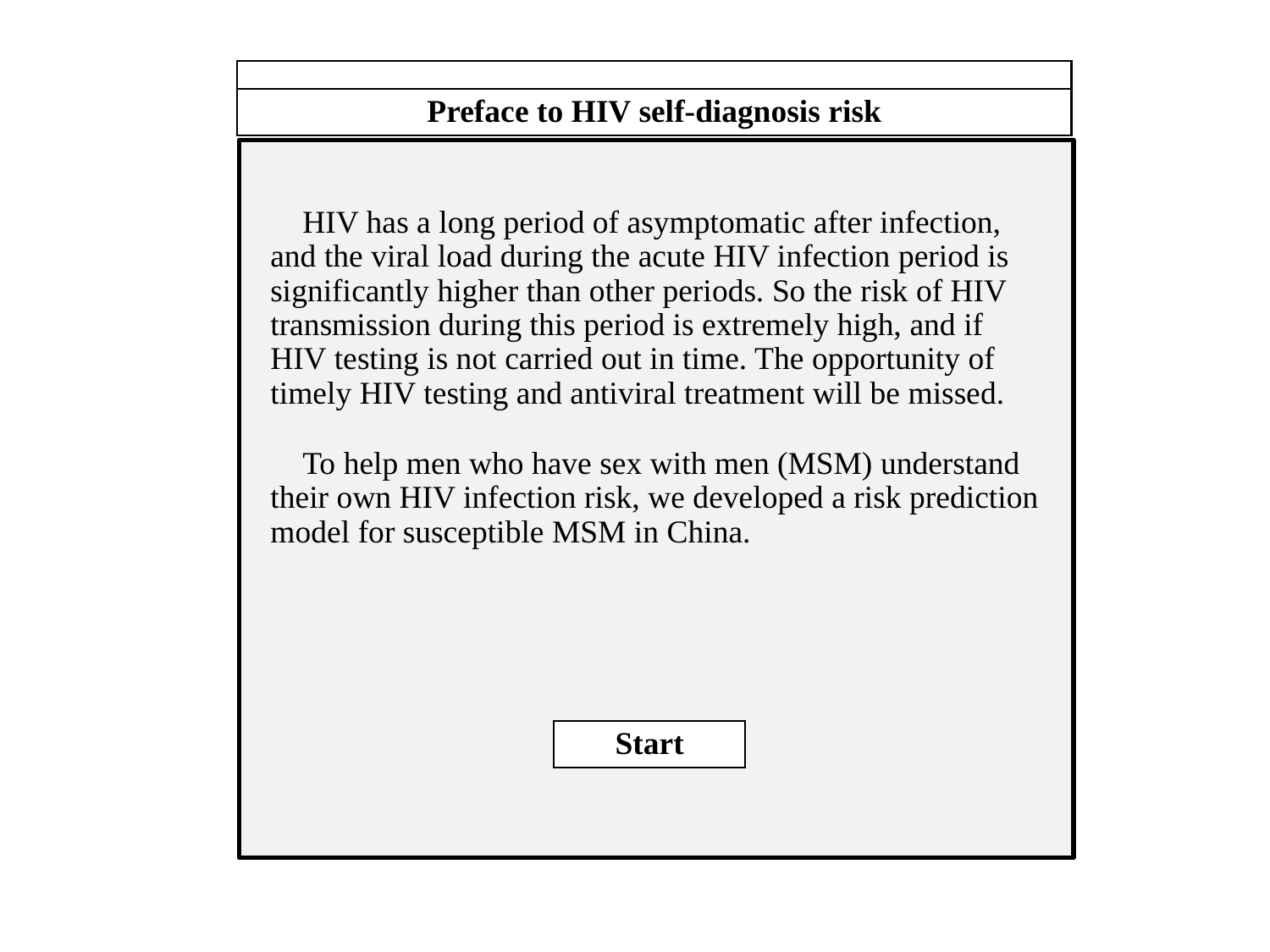

Preface to HIV self-diagnosis risk
 HIV has a long period of asymptomatic after infection, and the viral load during the acute HIV infection period is significantly higher than other periods. So the risk of HIV transmission during this period is extremely high, and if HIV testing is not carried out in time. The opportunity of timely HIV testing and antiviral treatment will be missed.
 To help men who have sex with men (MSM) understand their own HIV infection risk, we developed a risk prediction model for susceptible MSM in China.
Start

## Slide 5
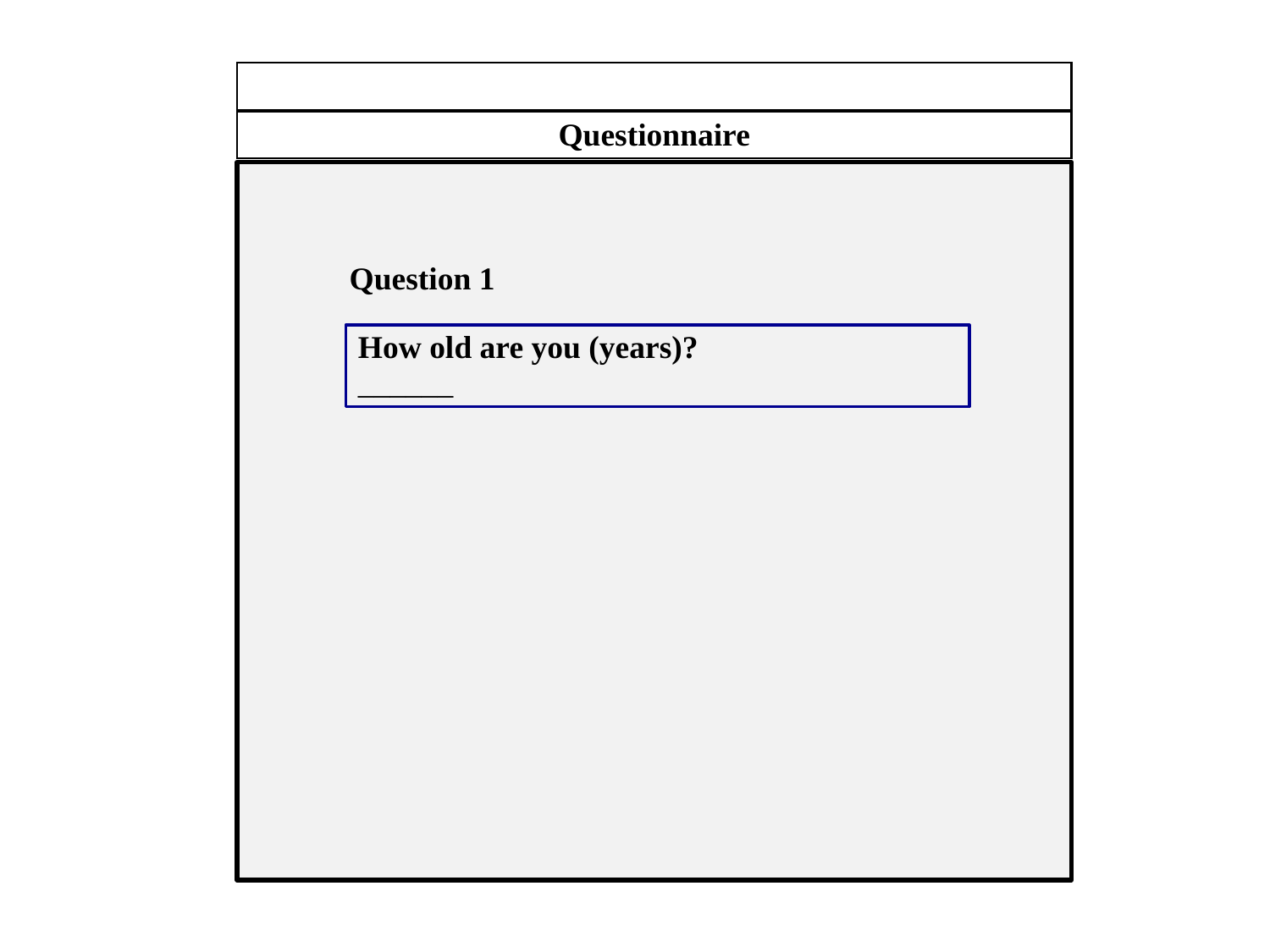

Questionnaire
Question 1
How old are you (years)?
______

## Slide 6
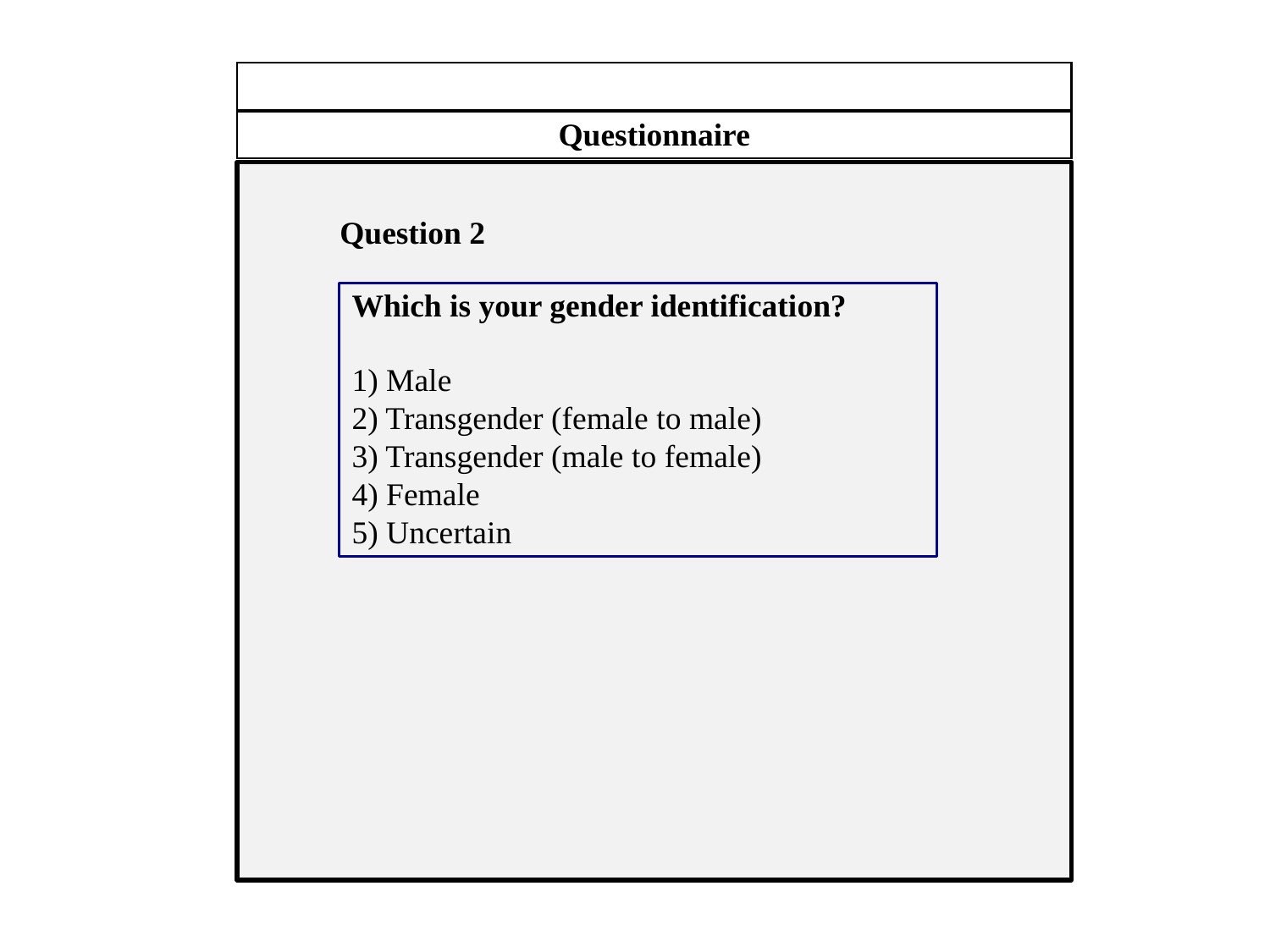

Questionnaire
Question 2
Which is your gender identification?
1) Male
2) Transgender (female to male)
3) Transgender (male to female)
4) Female
5) Uncertain

## Slide 7
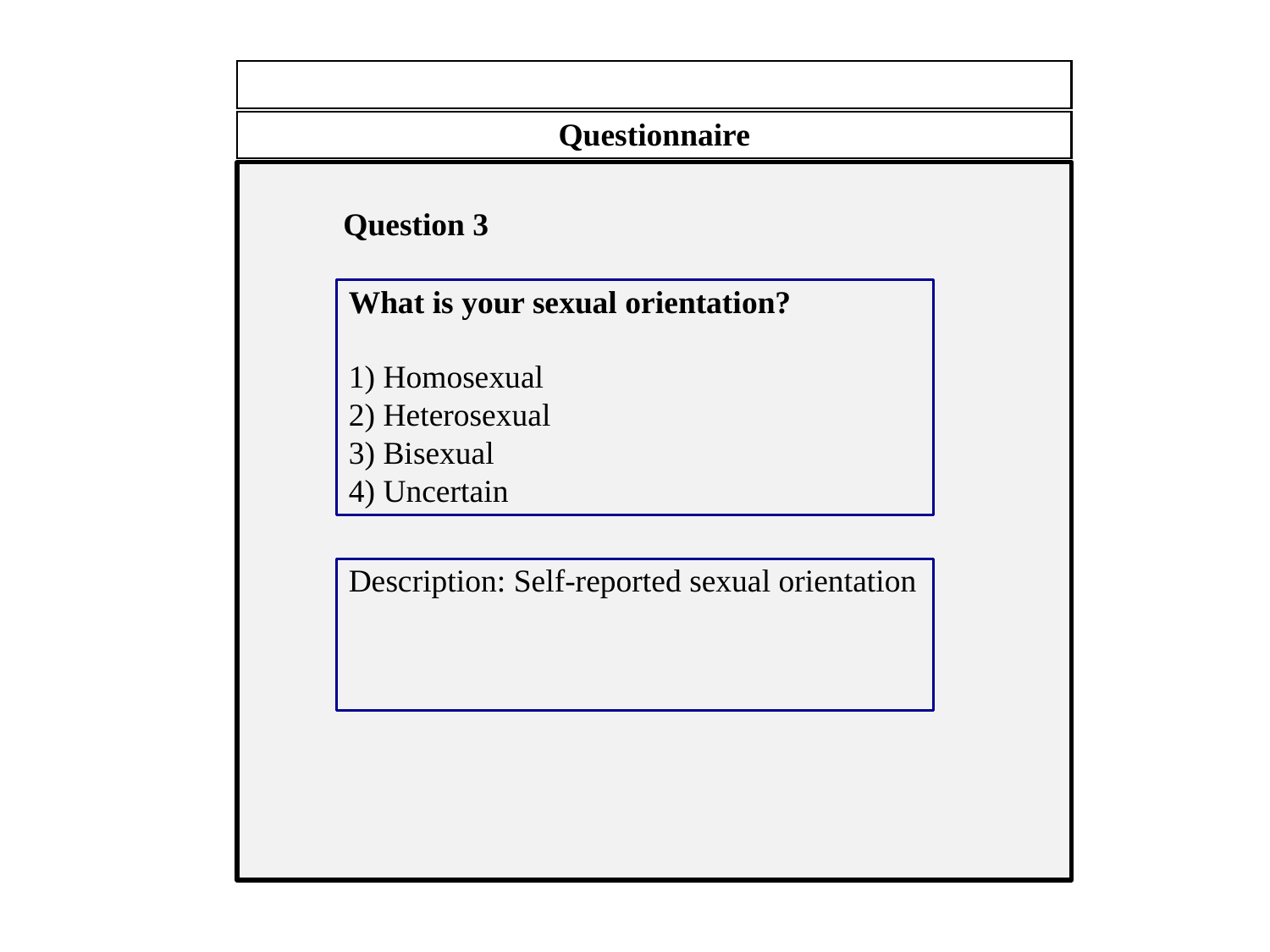

Questionnaire
Question 3
What is your sexual orientation?
1) Homosexual
2) Heterosexual
3) Bisexual
4) Uncertain
Description: Self-reported sexual orientation

## Slide 8
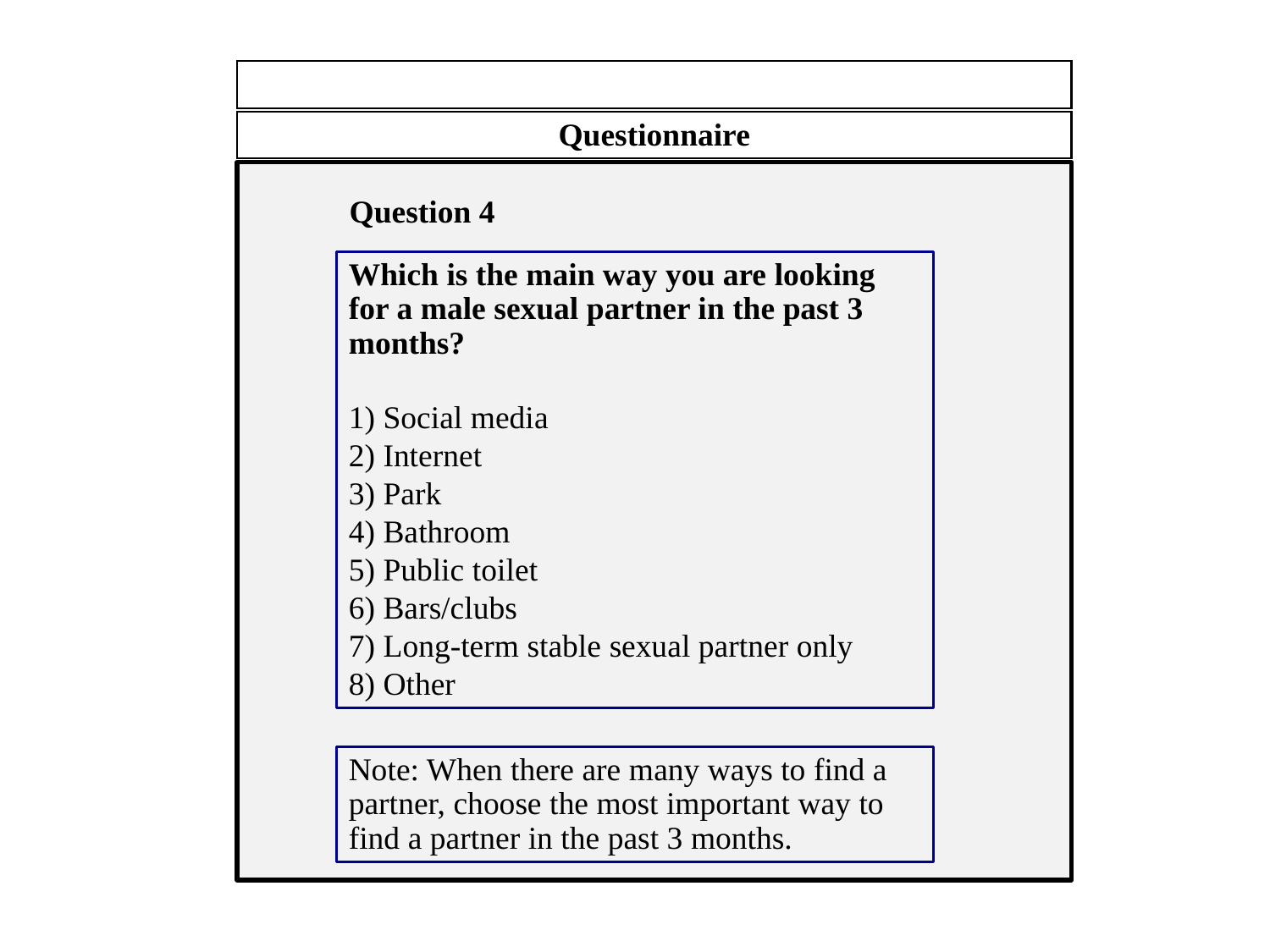

Questionnaire
Question 4
Which is the main way you are looking for a male sexual partner in the past 3 months?
1) Social media
2) Internet
3) Park
4) Bathroom
5) Public toilet
6) Bars/clubs
7) Long-term stable sexual partner only
8) Other
Note: When there are many ways to find a partner, choose the most important way to find a partner in the past 3 months.

## Slide 9
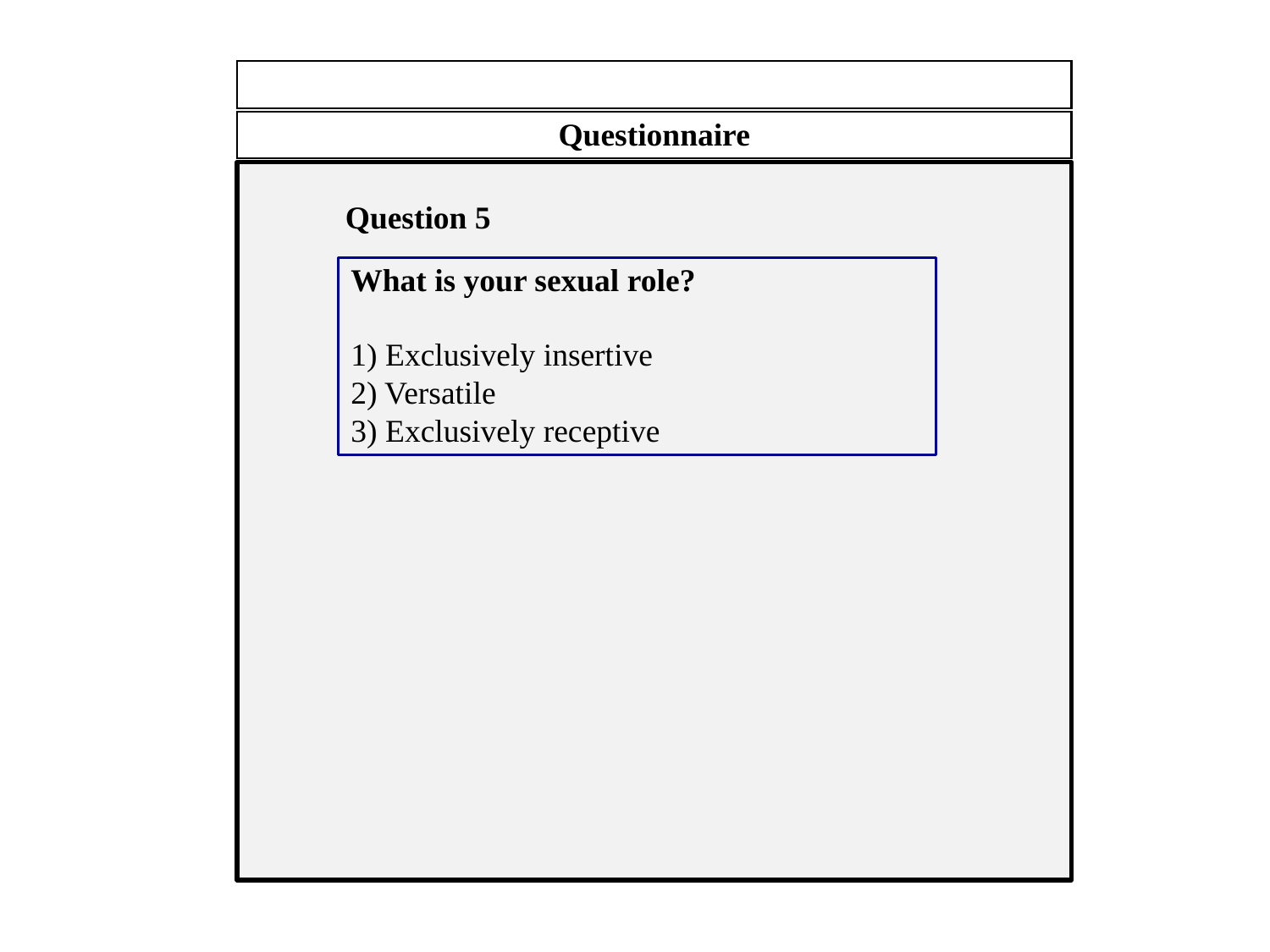

Questionnaire
Question 5
What is your sexual role?
1) Exclusively insertive
2) Versatile
3) Exclusively receptive

## Slide 10
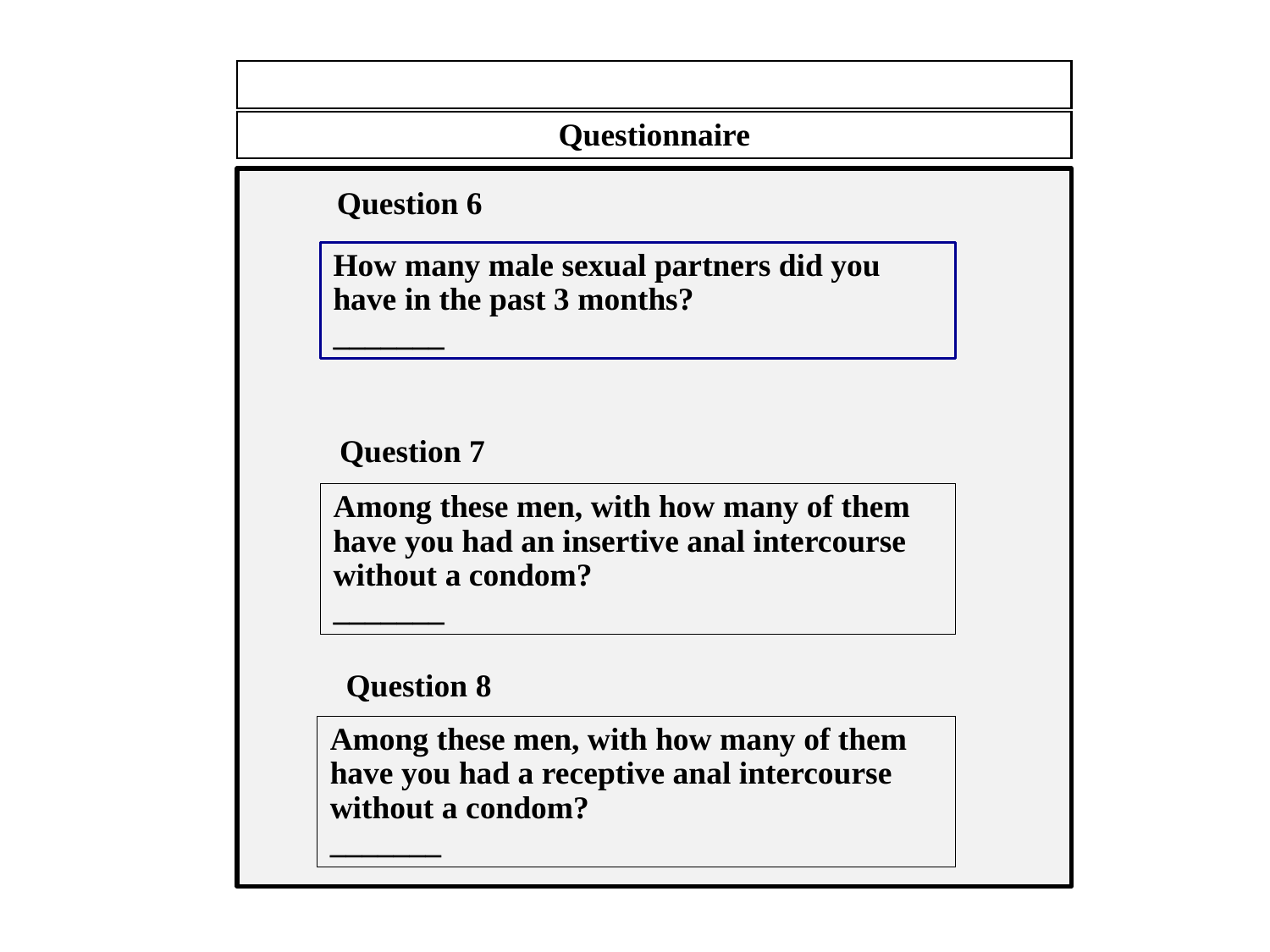

Questionnaire
Question 6
How many male sexual partners did you have in the past 3 months?
_______
Question 7
Among these men, with how many of them have you had an insertive anal intercourse without a condom?
_______
Question 8
Among these men, with how many of them have you had a receptive anal intercourse without a condom?
_______

## Slide 11
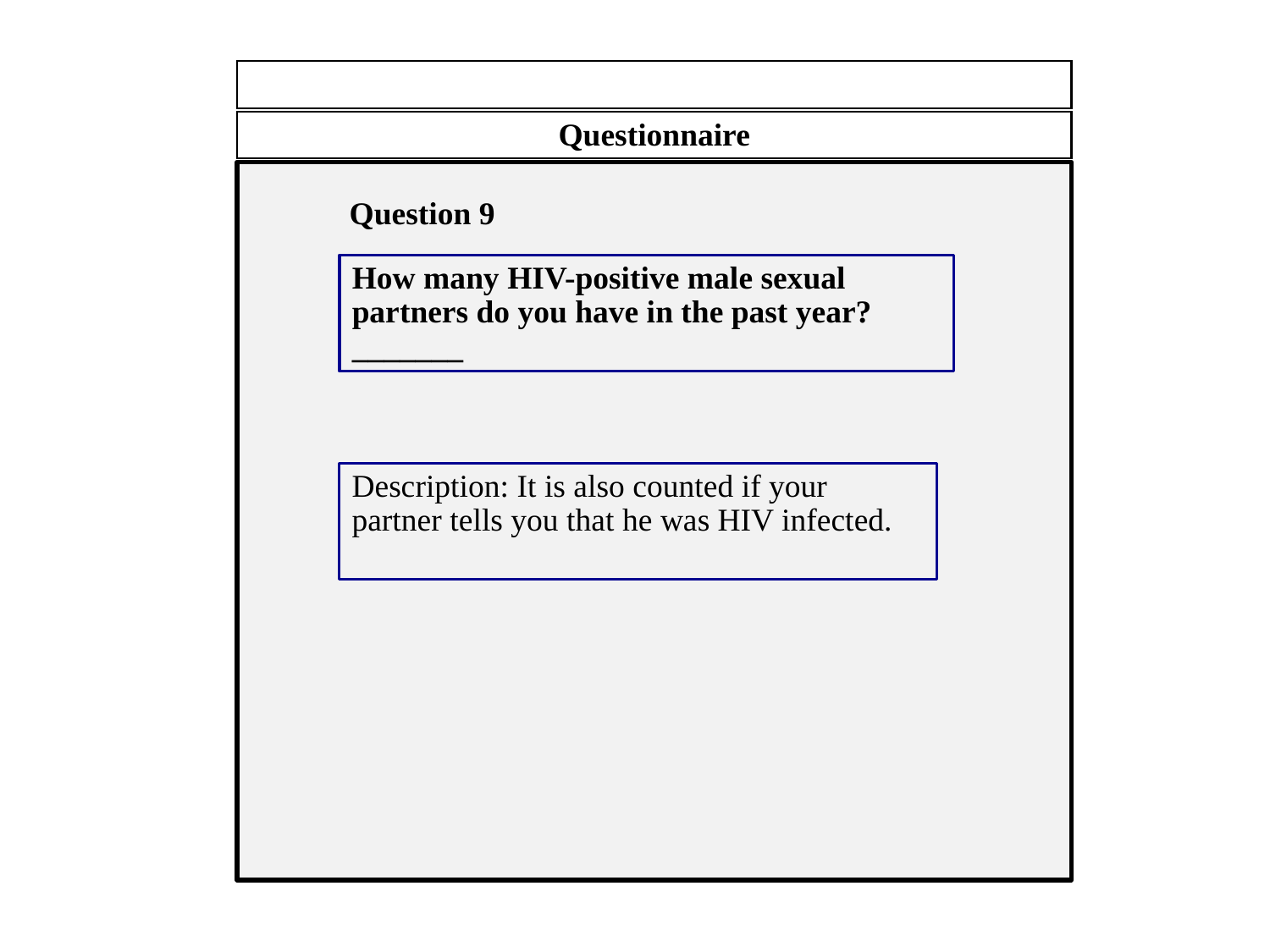

Questionnaire
Question 9
How many HIV-positive male sexual partners do you have in the past year?
_______
Description: It is also counted if your partner tells you that he was HIV infected.

## Slide 12
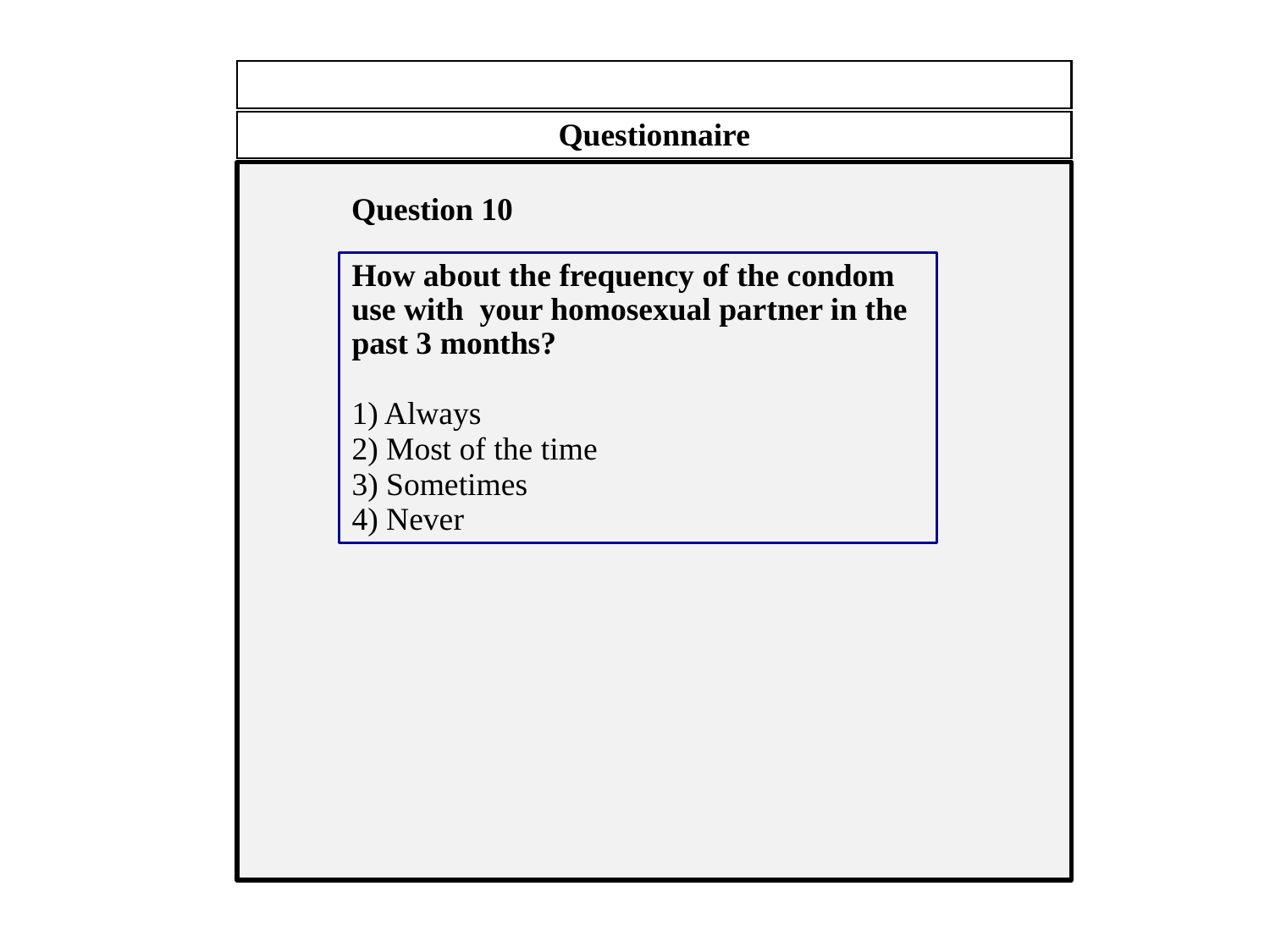

Questionnaire
Question 10
How about the frequency of the condom use with your homosexual partner in the past 3 months?
1) Always
2) Most of the time
3) Sometimes
4) Never

## Slide 13
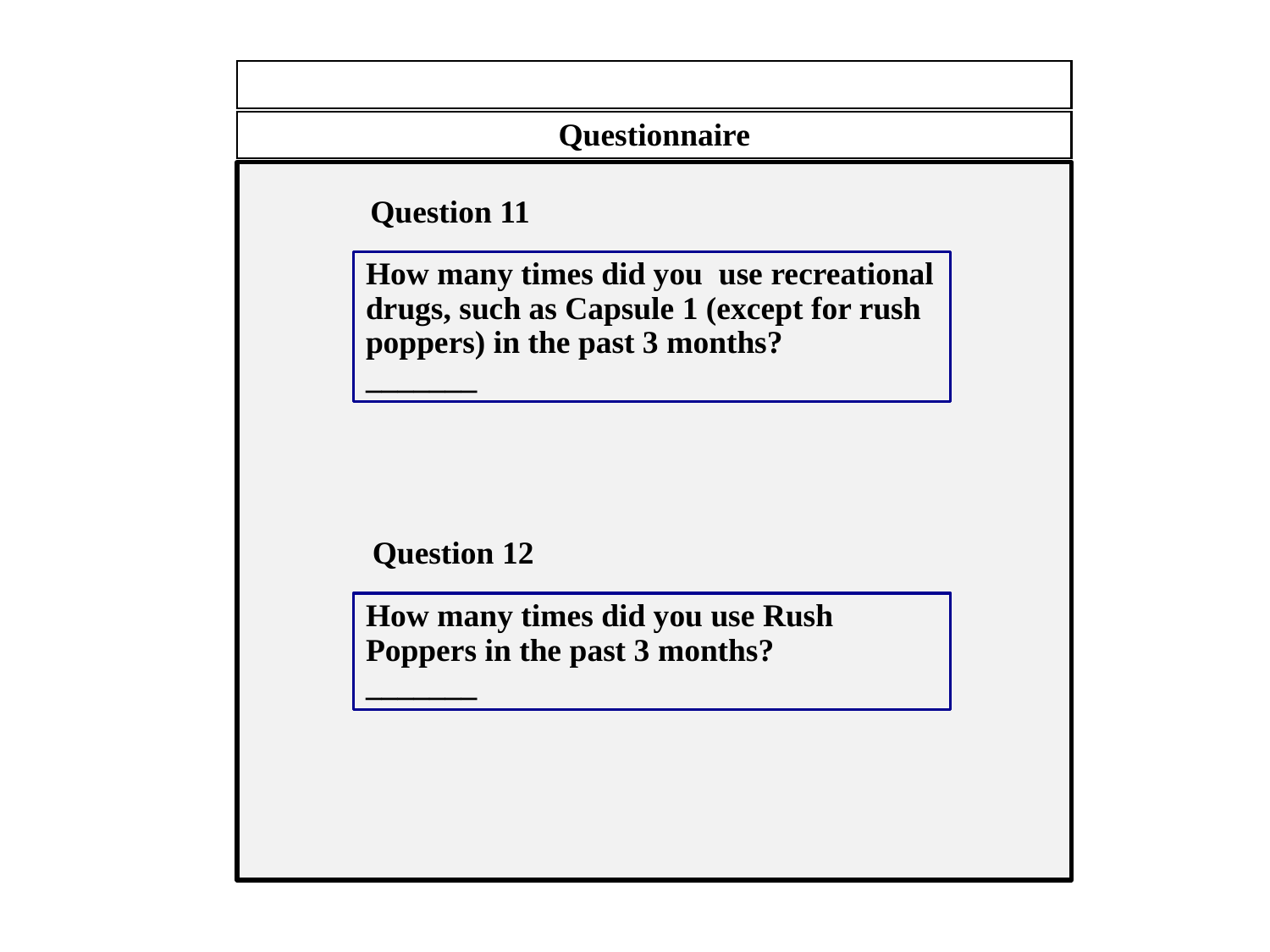

Questionnaire
Question 11
How many times did you use recreational drugs, such as Capsule 1 (except for rush poppers) in the past 3 months?
_______
Question 12
How many times did you use Rush Poppers in the past 3 months?
_______

## Slide 14
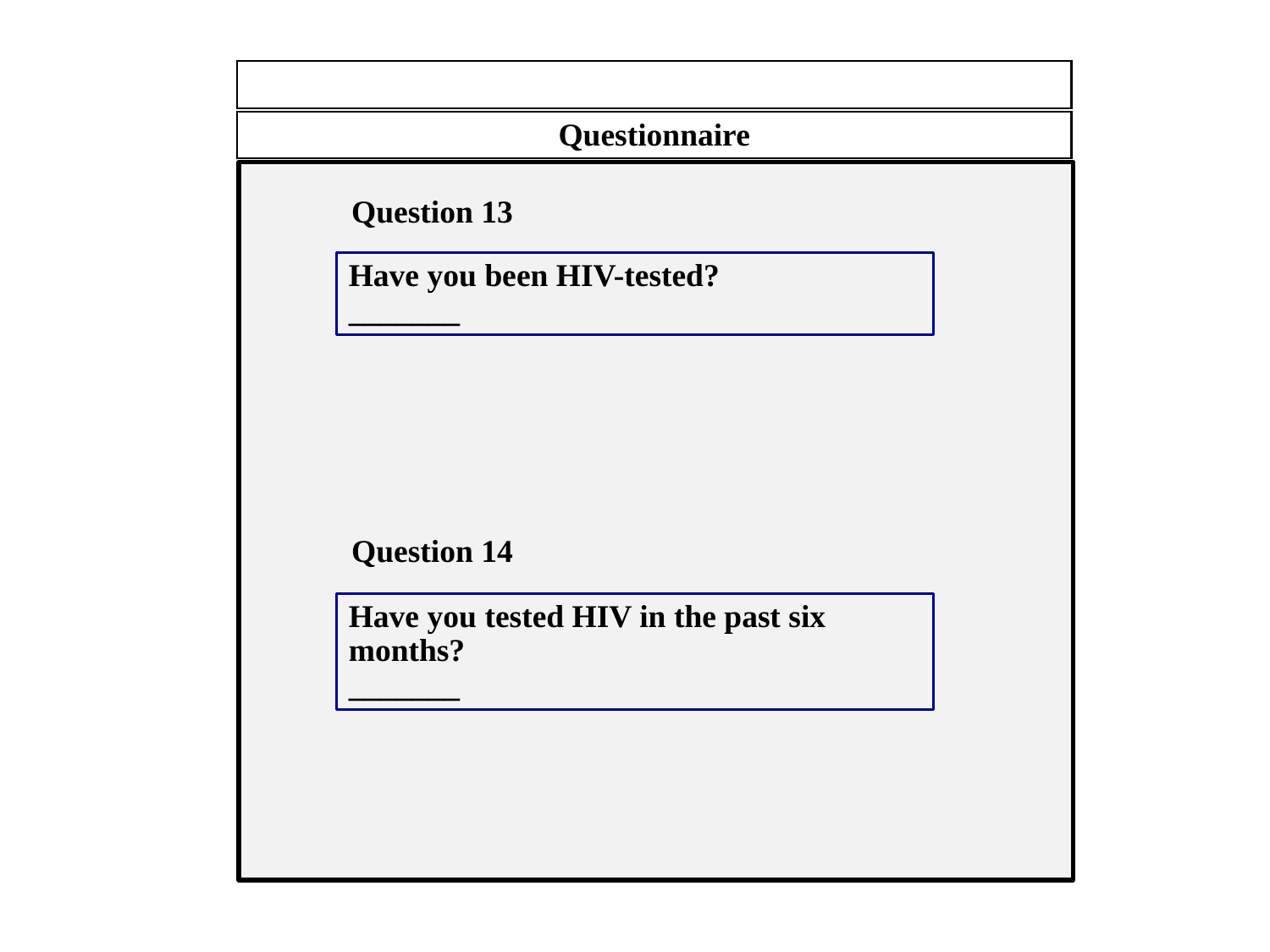

Questionnaire
Question 13
Have you been HIV-tested?
_______
Question 14
Have you tested HIV in the past six months?
_______

## Slide 15
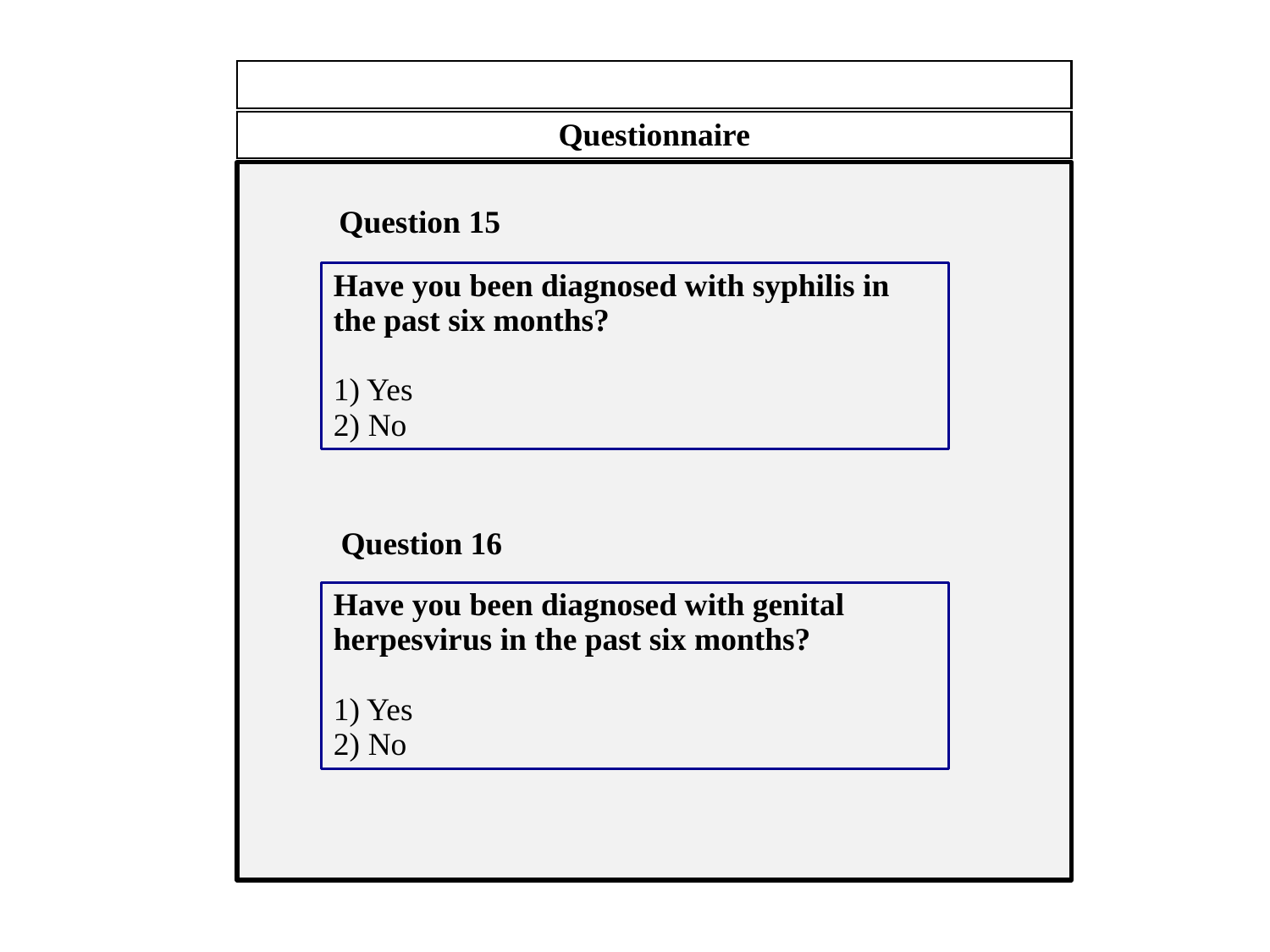

Questionnaire
Question 15
Have you been diagnosed with syphilis in the past six months?
1) Yes
2) No
Question 16
Have you been diagnosed with genital herpesvirus in the past six months?
1) Yes
2) No

## Slide 16
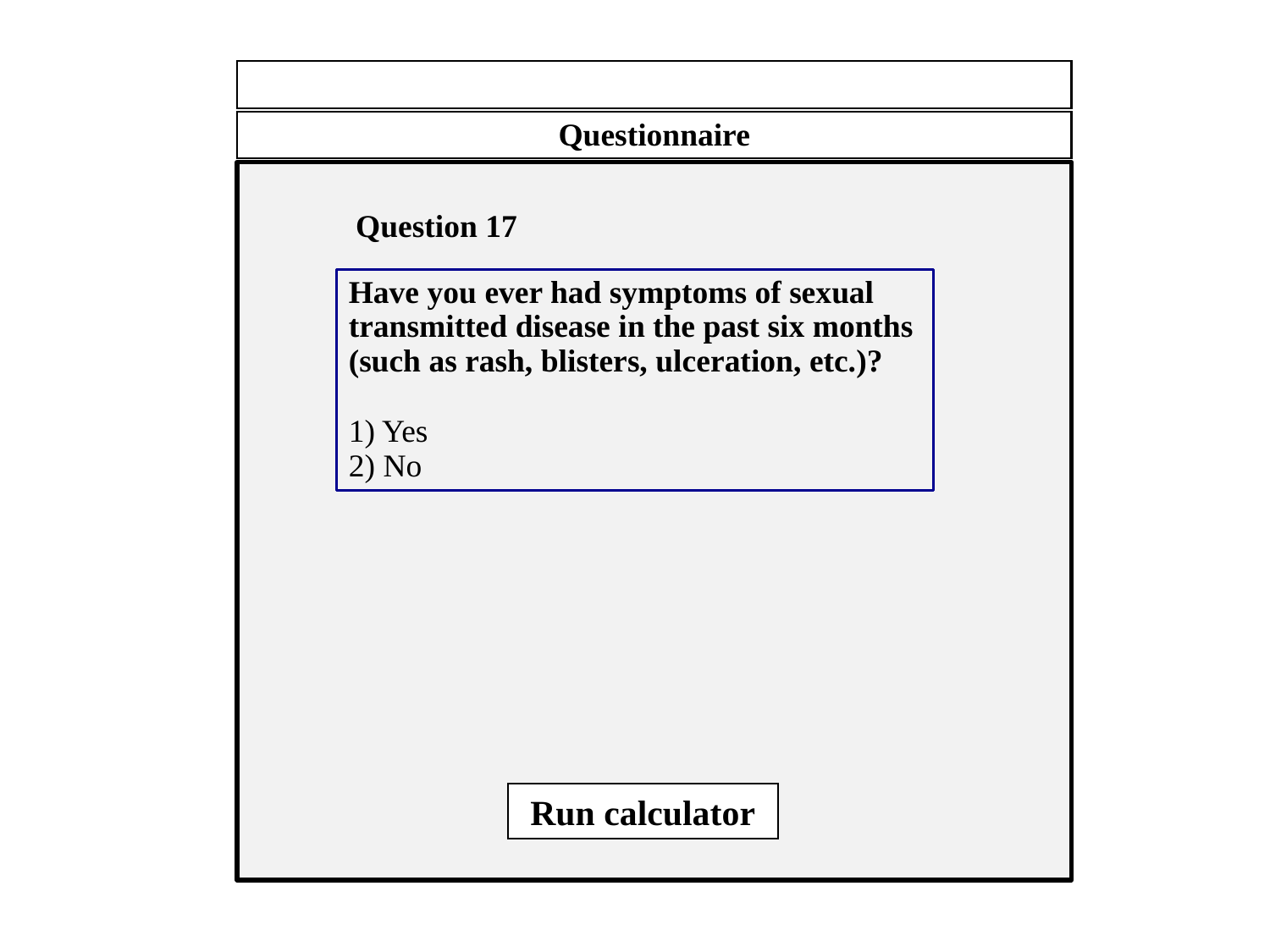

Questionnaire
Question 17
Have you ever had symptoms of sexual transmitted disease in the past six months (such as rash, blisters, ulceration, etc.)?
1) Yes
2) No
Run calculator

## Slide 17
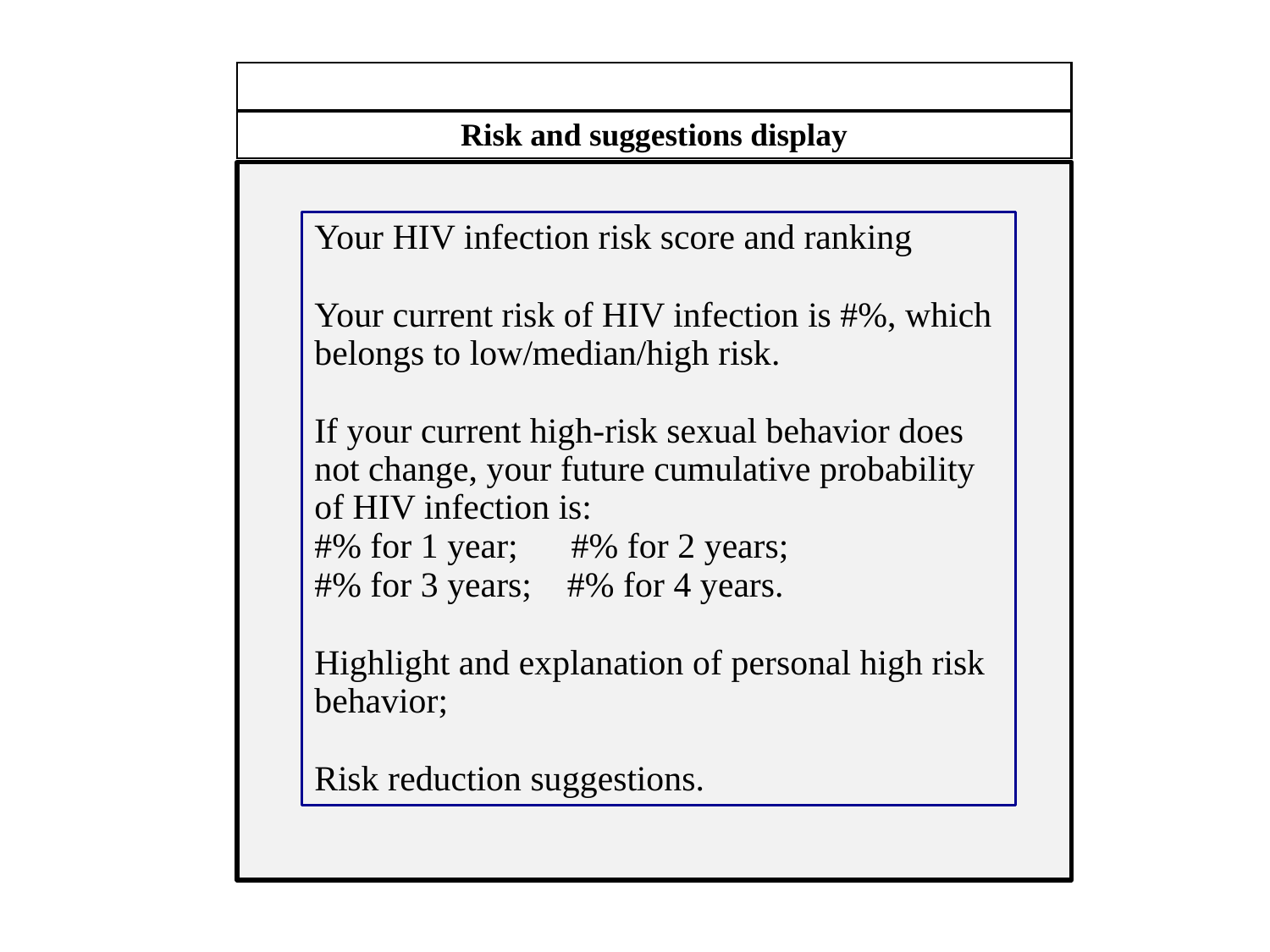

Risk and suggestions display
Your HIV infection risk score and ranking
Your current risk of HIV infection is #%, which belongs to low/median/high risk.
If your current high-risk sexual behavior does not change, your future cumulative probability of HIV infection is:
#% for 1 year; #% for 2 years;
#% for 3 years; #% for 4 years.
Highlight and explanation of personal high risk behavior;
Risk reduction suggestions.
